# Supplementary material for: Standardization of enzyme-linked immunosorbent assays for serosurveys of the SARS-CoV-2 pandemic using clinical and at-home blood sampling
Source: medRxiv. 2020 May 25:2020.05.21.20109280. Preprint. [Version 1] doi: 10.1101/2020.05.21.20109280 (PMC7265693; doi:10.1101/2020.05.21.20109280)
Supplement: Supplement 2020 [file 82602-2020.05.21.20109280-1.pdf]

Supplemental Materials: Klumpp-Thomas et al. 2020

SUPPLEMENTARY MATERIALS AND METHODS

Semi-Automation of ELISA

The BioTek Instruments EL406 washer/dispenser used is equipped with various liquid handling capabilities including 2 syringe pump dispense heads with 16 nozzles each, a peristaltic pump dispense option which can be outfitted with an 8-tip bulk dispense cassette and, a 96-channel wash head for aspirating and dispensing. The unit is outfitted with a 30-plate stacker to input plates, returning them to the output stack when finished with a protocol step as well as a restack function to return the plates to the original order. There are four 4 L bottles which can be used to dispense from and clean the 96-channel wash head using a valve module to switch between the different source bottles. Two waste collection vessels, a primary as well as a secondary containing a liquid level sensor to alert when emptying is necessary are also plumbed to the washer/dispenser. The unit used for these experiments has an integrated BioStack 4 microplate stacker, but this is not necessary to have in order to perform the described protocol; loading the plates manually onto the BioTek EL406 plate nest will yield the same liquid handling results. It is important to note that the aspiration heights described within this process were optimized for the particular 96-well plate used in this ELISA protocol and would need to be re-optimized if a different assay plate was used.

This process will only describe semi-automated steps and will not detail manual segments. (1) 96-well plates are currently coated with the Spike or RBD protein manually using a multichannel pipette but alternatively the peristaltic 8-tip small volume dispense cassette on the BioTek EL406 will be incorporated in the future. (2) After 16 hours of incubation at 4°C the plates are loaded into the input stack of the washer/dispenser, the syringe B dispense line is primed with blocking solution, and the 96-channel wash head is primed with PBST wash solution. Prior to each step, priming the associated dispense lines and wash head must occur. (3 + 4) The blocking protocol is run for the loaded plates which entails washing all wells three times (3x) with PBST followed by dispensing 200 µL of blocking solution. In subsequent steps, 'washing' refers specifically to aspirating the existing liquid, in this case coating protein, from the well and dispensing 300 µL of PBST into the same wells using the 96-channel wash head on the BioTek EL406 washer/dispenser. The height of the aspiration pins for the first two wash cycles is 3.68 mm above the BioTek plate carrier and the last aspiration happens slightly lower at a height of 3.43 mm above the plate carrier with an additional secondary aspirate at the same height 2.06 mm to the left of the well center in order to remove the remaining liquid on the well perimeter prior to blocking solution addition. For

reference, well bottom is roughly 3.4 mm above the plate carrier. Once all plates have been washed with block added into the wells and returned to the output stack, all plates are then restacked into the original order into the input stack.

It is necessary to clean the syringe B dispense head and line soon after the block has been added to all plates to prevent clogging of the dispense tips. Each time a dispense happens throughout this protocol it will be assumed that the following cleaning protocol has been performed for the syringe head used. The bottle cap with the liquid sourcing end of line is fastened into an empty bottle and primed with 8 mL of air two times sequentially, next the same cap and line are secured into a bottle containing deionized water and primed with 8 mL five times, a clean empty bottle is then hooked up again and primed with 8 mL of air two times, next the cap and line are placed onto a bottle containing 70% ethanol and primed five consecutive times with 8 mL, followed up again with two 8 mL primes of air and a final deionized water rinse priming 8 mL five times. Before a new reagent is primed through the respective line two primes of 8 mL using a clean empty bottle is completed for separation of water and new reagent.

(5) After the 2 hour incubation (6) the plates are washed 3x with PBST to remove blocking solution and roughly 30 µL is left in all wells to prevent drying before sample addition. Here on out, it will be assumed that incubation steps happening at room temperature will be stacked in the BioTek input stacker with a lid, which will be removed before beginning next step, on the top plate. The height of the aspiration pins for the first two wash cycles is 3.68 mm above the carrier and the final aspiration happens at 4.70 mm above the plate carrier in order to retain some wash fluid as mentioned. (7) Currently samples have been added to the ELISA plates using a multichannel pipette; for the scaled-up process an Integra Biosciences VIAFLO electronic 96 channel pipette can be used to transfer 100 µL of the samples from a 96 deep-well block into multiple ELISA destination plates.

(8) Once the 1 hour incubation ends (9 + 10) all wells in the plates within the associated batch will be washed 3x with PBST followed by the dispensing 100 µL of the respective antibody into all wells of the 96-well plate using the BioTek EL406 washer/dispenser. The height of the aspiration pins for all three wash cycles is at 3.43 mm above the plate carrier and also include a secondary aspiration at the same height 2.06 mm to the left of well center. Each addition of PBST wash fluid also incorporates a 15 second soak prior to aspiration. This washing procedure is critical to decreasing background and fully removing the samples (primary antibody). Plates are restacked into the original order within input stack.

(11) After the 1 hour incubation, (12) all wells in the batch of plates are washed 2x with PBST and the final dispense of 300 μL is added using the 96-channel wash head. The height of the aspiration pins for these two cycles is 3.68 mm above the carrier, also includes a secondary aspiration at the same height 2.06 mm to the left of well center, and each addition of PBST wash fluid incorporates a 25 second soak prior to aspiration. The difference in soak time from the previous process of washing the samples out of the wells allows the timing to stay consistent for each assay plate. Plates are then restacked into the original order within the input stack with the remaining 300 μL of PBST.

Performing the initial two washes with PBST and leaving the last 300 μL in the wells enables the addition of substrate to happen quicker than if all three washes were to happen prior to substrate addition. With the critical nature of the 10 minute incubation for substrate and to increase throughput, it is desired to systematically increase the speed of substrate addition as in this protocol.

(13) The remaining PBST is removed from all wells of the plates using the BioTek 96-channel wash head. A height of 3.43 mm above the plate carrier is set for the aspiration pins and a secondary aspirate at the same height 2.06 mm to the left of well center is performed. A volume of 100 μL of substrate is added to all wells using syringe A dispense head which is the designated line used for substrate only as it is a sensitive reagent. As plates are dispensed as loaded from the input stack they are returned to the output stack and after the last plate is finished all plates are restacked in the original order within the input stack. (14) Each plate is incubated with the substrate solution for 10 minutes.

(15) It is critical that the stop solution is added after the 10 minute incubation, therefore the plate batch size at this step is adjusted accordingly. As soon as the first plate has finished incubating with substrate the Biotek protocol for adding 100 μL of the stop solution using syringe B dispense head is started. An important note regarding the stop solution used in this ELISA protocol is that when 100 μL was set for the dispensing variable approximately 60 μL was actually added to the wells. In order to compensate for the characteristics of this liquid 175 μL was set as the dispense variable for the stop solution addition. It will be checked regularly for volume accuracy. Once all plates have received stop solution they are restacked into the original order into the input stack.

(16) Absorbance is measured at 450 nm and 650 nm. Data was initially collected using the BMG Labtech PHERAstar FSX, and the BioTek Epoch 2 has subsequently been used. The BioTek stacker containing the plates which have been dispensed with stop solution and returned to the original order is removed from the BioTek input stack position and placed into the input stack position for the Epoch 2. The read protocol is

started, and plates are fed into the Epoch 2 for data collection. The BioTek EL406 96-channel wash head is cleaned at the end of each day to ensure no reagent buildup or clogging happens. This entails flushing 350 mL of deionized water followed by 350 mL of 70% ethanol and finally with 350 mL of deionized water. The 96-channel wash head dispense tips as well as the aspiration pins are cleaned in this process.

Based on thorough titering studies of both archival and PCR+ SARS-CoV-2 diagnosed patient sera (**Supplementary Figure 7**), we determined that the optimal titrations for the sample into ELISA were 1:400 to 1:1000 for IgG, and 1:400 for IgM and IgA (as IgM and IgA are at lower quantities in the blood when compared to IgG). Microsampler dilution was adjusted accordingly to 1:10 for IgM/IgA and 1:25 for IgG into PBS + 5.0% NFDM, or PBS + 0.03% Tween20, respectively, to ultimately result in 0.05% Tween20 in the final diluted sample for both 1:10 and 1:25 dilutions.

Statistical Modeling

Let r_1, r_2 be the values from the census for proportion of people in each population. Let n be the number of people in the sample. Let y_{1i} be the ith sampled response and n_1 the number ofobservations from population 1 and y_{2i} be the ith sampled response and n_2 the number of observations from population 2. The weights for an observation in population 1 or 2 are $w_1 = \frac{r_1}{n_1/n}$ and $w_2 = \frac{r_2}{n2/n}$. The unadjusted estimate of the prevalence is the weighted estimate of the mean, which is

$$\tilde{p} = \frac{\sum_{i=1}^{n_1} w_1\, y_{1i} + \sum_{i=1}^{n_2} w_2\, y_{2i}}{\sum_{i=1}^{2} \sum_{j=1}^{n_i} w_i} = \frac{n_1 w_1 \hat{p}_1 + n_2 w_2 \hat{p}_2}{n}$$

where $\hat{p}_i$ are the observed proportions in each population. The variance of $\tilde{p}$ is

$$\widehat{var}(\tilde{p}) = \frac{n_1 w_1^2 \hat{p}_1 (1 - \hat{p}_1) + n_2 w_2^2 \hat{p}_2 (1 - \hat{p}_2)}{n^2}$$

Lang and Reiczigel[1] (2014) provide a prevalance estimate that adjusts for the sensitivity and specificity of the assay, taking into account the variability of the estimate of prevalence as well as the variability in the estimation of sensitivity and specificity. Thus, the Lang-Reiczigel method requires the sample sizes for validation of the assay, specifically, the number of true positives used to estimate the sensitivity, and the number of true negatives used to estimate the specificity. Although the method was developed for simple random samples, we make the following modification for other survey designs. Let $\tilde{p}$ and $\widehat{var}(\tilde{p})$ be the prevalence and variance estimates under the design with perfect sensitivity and specificity. We wish to the know the sample size for the estimate of $\tilde{p}$ that would equal the variance of $\widehat{var}(\tilde{p})$. Therefore set

$$\widehat{var}(\tilde{p}) = \frac{\tilde{p}(1-\tilde{p})}{n_{eff}},$$

where the righthand side of the equation is modeled after the binomial variance from a simple random sample. In other words, the effective sample size is the simple random sample size that would give similar variance to the variance of the weighted design. Then we solve for n_{eff} so

$$n_{eff} = \frac{\tilde{p}(1-\tilde{p})}{\widehat{var}(\tilde{p})}$$

Then we input $\tilde{p}$ and n_{eff} into the Lang-Reiczigel method in place of the simple random sample prevalence estimate, $\hat{p}$, and its sample size, n.

Here are the Lang-Reiczigel equations with those modifications[1]. The estimate of prevalence adjusted for sensitivity and specificity is

$$\hat{\phi} = \frac{\tilde{p}' + S_p' - 1}{S_e' + S_p' - 1}$$

with $n_{eff}' = n_{eff} + z^2$, $\tilde{p}' = \frac{n_{eff}\tilde{p} + z^2/2}{n_{eff}'}$, $n_{Sp}' = n_{Sp} + 2$, $Sp' = \frac{n_{Sp}Sp+1}{n_{Sp}+2}$, $Se' = \frac{n_{Se}Se+1}{n_{Se}+2}$ with Sp and Se being the estimates of specificity and sensitivity, and n_{Sp} and n_{Se} being the number of observations used to estimate specificity and sensitivity, with z being the upper quantile from the standard normal distribution with probability 1-$\alpha/2$, where $\alpha = 1 - q$ and q is the confidence level for the confidence interval (e.g., 95%). Then

$$\widehat{var}(prev) = \frac{\tilde{p}'(1-\tilde{p}')/n_{eff}' + \hat{\phi}^2 * Se'(1-Se')/n_{Se}' + (1-\hat{\phi})^2 Sp'(1-Sp')/n_{Sp}')}{(Se' + Sp' - 1)^2}.$$

The estimate of the confidence intervals are given by

$$\hat{\phi} + k \pm z\sqrt{\widehat{var}(prev)}$$

with

$$k = 2z^2\left[\frac{\hat{\phi}Se'(1-Se')}{n_{Se}'} - \frac{(1-\hat{\phi})Sp'(1-Sp')}{n_{Sp}'}\right].$$

SUPPLEMENTAL REFERENCES

[1]Lang, Z. and Reiczigel, J., 2014. Confidence limits for prevalence of disease adjusted for estimated sensitivity and specificity. Preventive veterinary medicine, 113(1), pp.13-22. *(Note: In this reference, there is a typo in equation 4 of Lang and Reiczigel that defines* $\widehat{var}(prev)$*, in our notation the typo is that the* $(1-\hat{\phi})^2$ *expression was mistakenly written as* $(1+\hat{\phi})^2$*.)*

SUPPLEMENTAL FIGURES AND LEGENDS

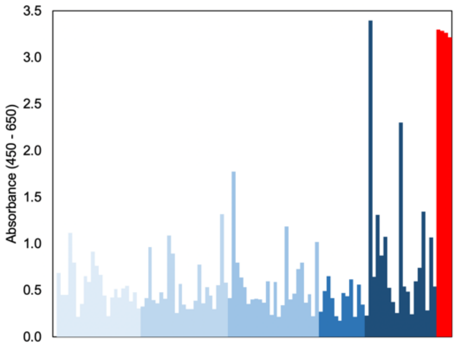

Supplementary Figure 1: Archival negative control samples at 1:100 dilution. High background observed at 1:100 dilution of sera into ELISA.

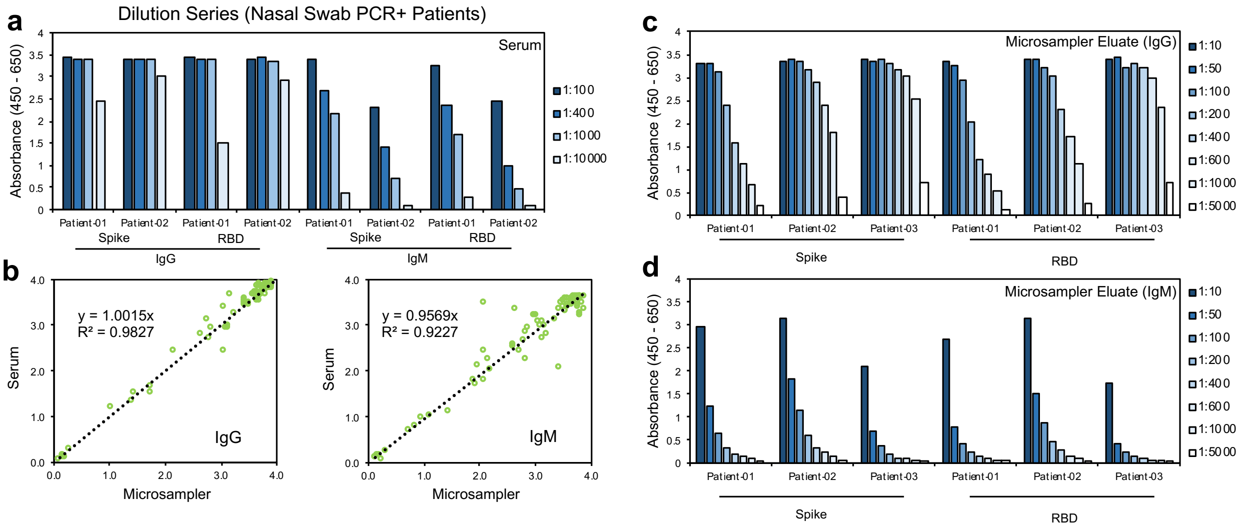

Supplementary Figure 2: Titering of serum and microsampler eluate in manual ELISA. (a) Dilution series of serum from PCR+ diagnosed patients for both IgG and IgM. (b) Linear correlation between microsampler eluate and serum from the same donor (n = 68 donors from high-exposure community, microsampler dilution = 1:10, estimated final 1:400 serum dilution equivalent based on 50% serum volume in blood, serum dilution = 1:400) (c) IgG dilution series of microsamplers from PCR+ diagnosed patients. (d) IgM dilution series of microsamplers from PCR+ diagnosed patients.

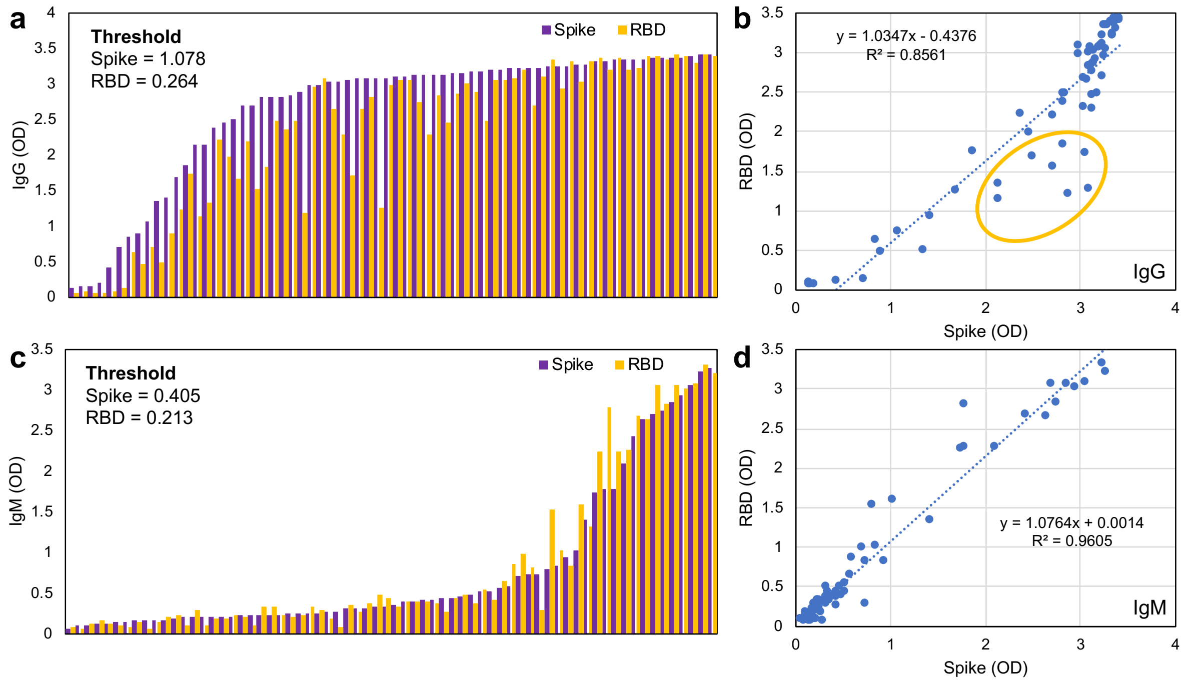

Supplementary Figure 3: Comparison of Spike and RBD signal intensity for IgG and IgM in high seroprevalence community. (**a**) IgG absorbance (OD) displayed as increasing Spike (purple) OD compared to RBD (yellow). (**b**) Correlation between Spike and RBD IgG signal intensity. (**c-d**) Spike & RBD IgM signal intensity and correlation.

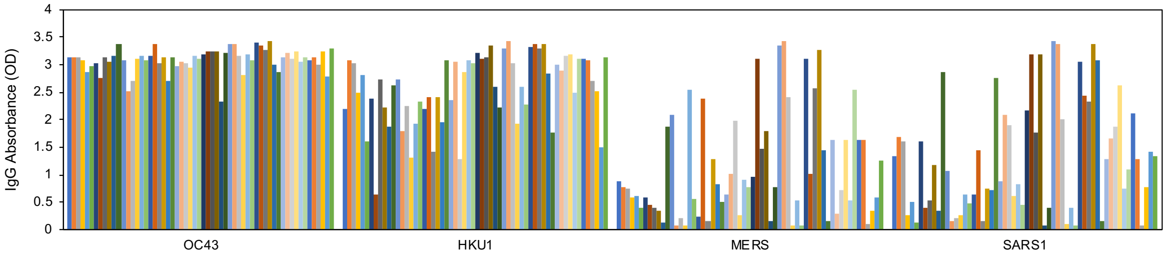

Supplementary Figure 4: Raw absorbance data of OC43, HKU1, MERS, and SARS1 for hard-hit test data set. IgG absorbance for OC43, HKU1, MERS and SARS1.

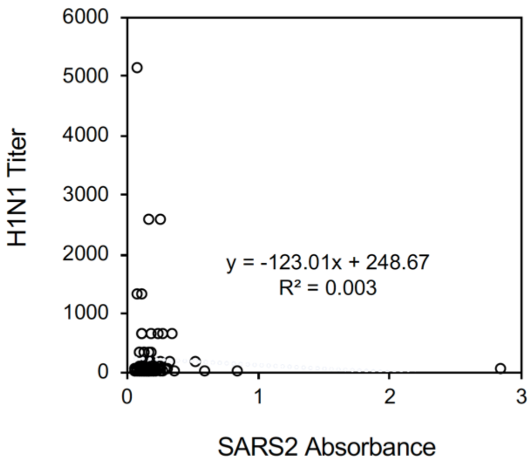

Supplementary Figure 5: H1N1 Titer as a function of SARS-2 absorbance shows no correlation between influenza titer and SARS2 signal intensity

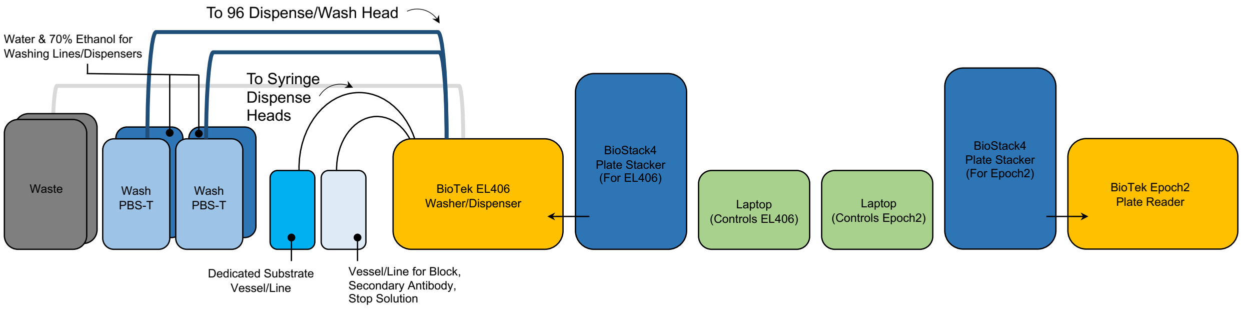

Supplementary Figure 6: Physical setup of semi-automated ELISA platform.

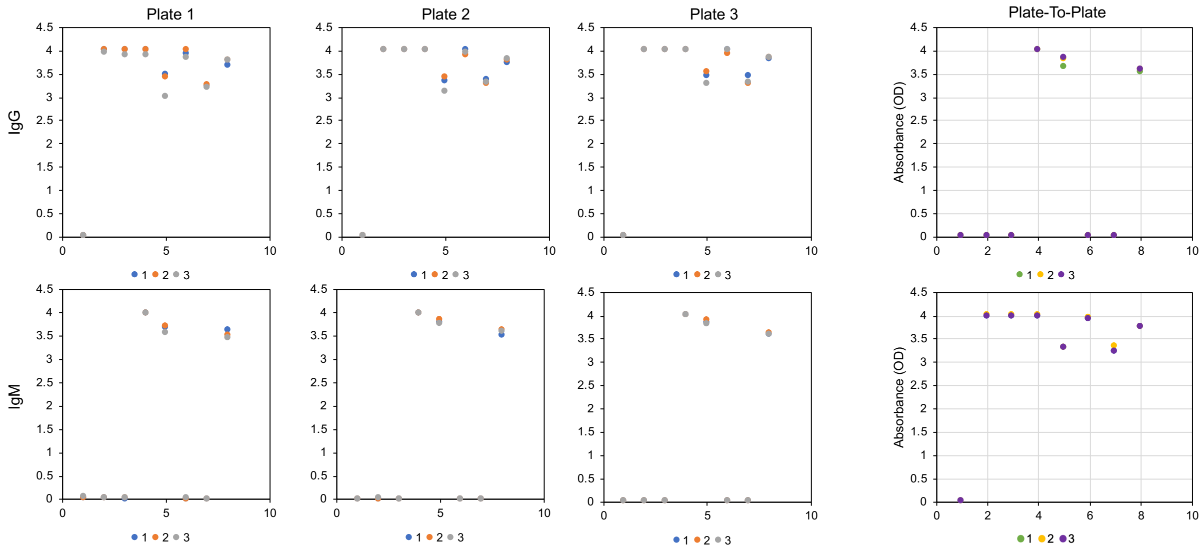

Supplementary Figure 7: Technical repeatability from well-to-well and plate-to-plate in IgG and IgM ELISA using a semi-automated setup.

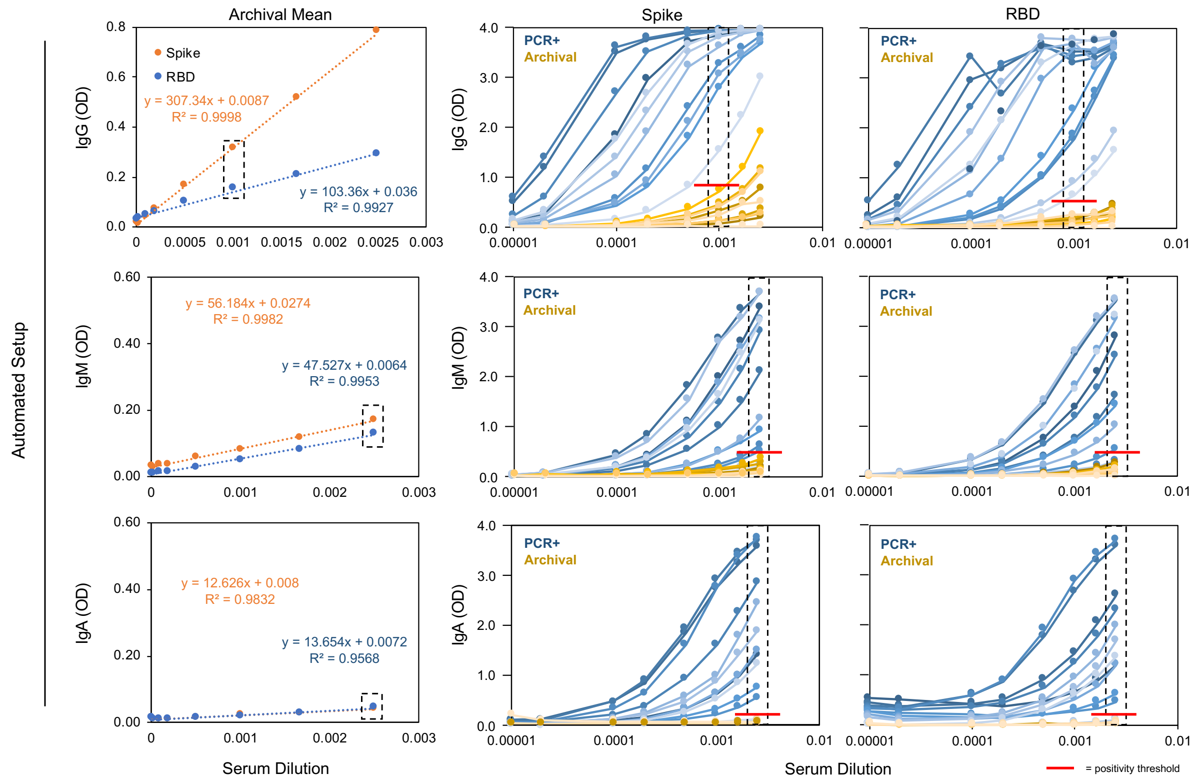

Supplementary Figure 8: Evaluation of optimal titer for automated setup. (a) Average (n = 8) archival serum signal intensity at multiple dilution titers. Dashed square surrounding lowest titer suggested to be used. (b) Titers of PCR+ confirmed patients (*n* = 11) for spike (left) and RBD (right). Red line = threshold of lowest titer suggested to be used.

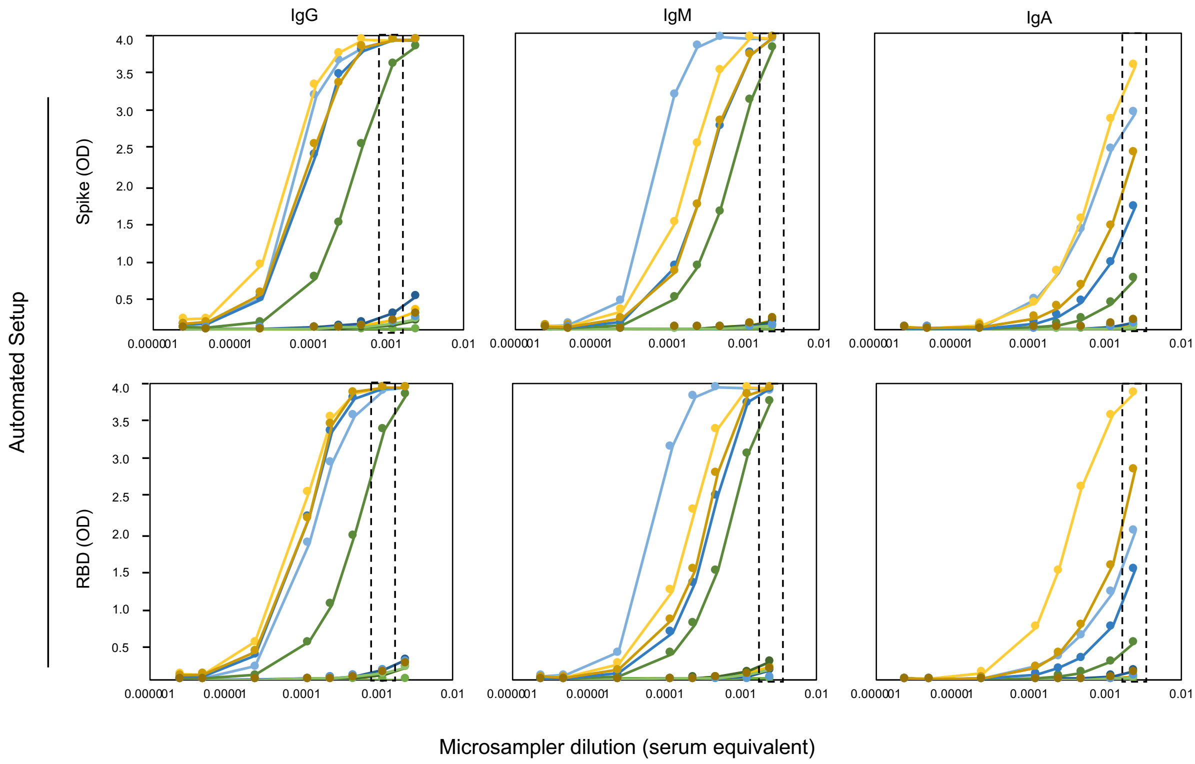

Supplementary Figure 9: Confirmation of dilution using microsamplers from high prevalence small test cohort. Titers for spike and RBD, for IgG, IgM and IgA. Square around selected titer. Titers displayed as estimate serum titer (assuming 10ul serum volume on 20ul blood microsampler and initial 1:40 dilution into elution buffer).

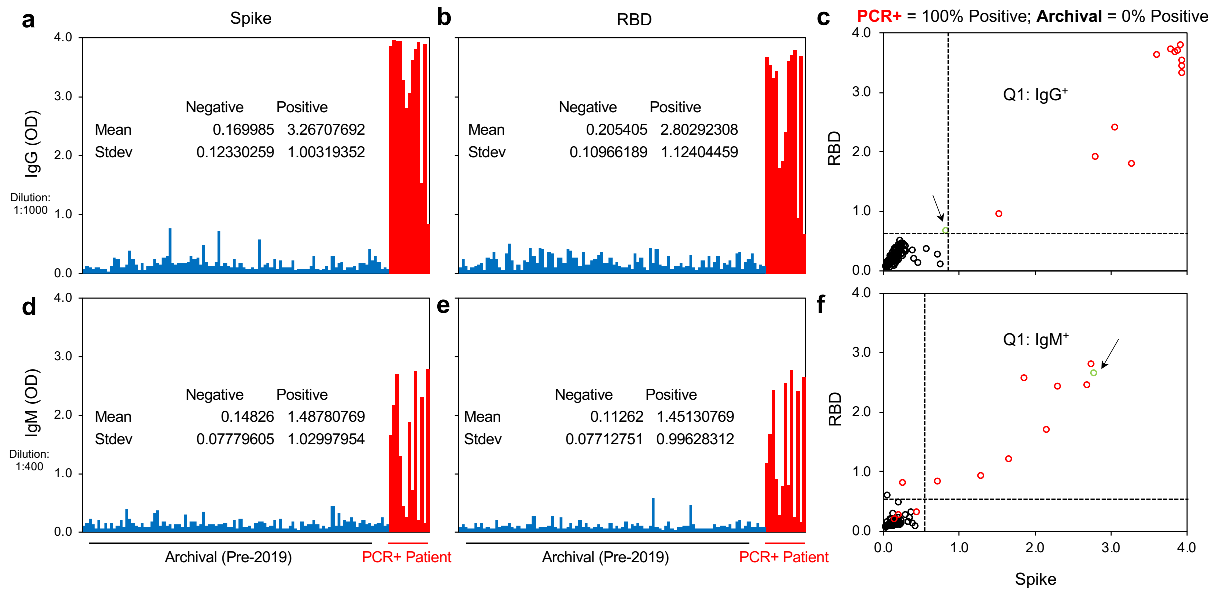

Supplementary Figure 10: Archival negative controls re-validated on semi-automated setup. (a) Spike IgG, (b) RBD IgG, (c) Threshold displayed for IgG seropositive determination. (d) Spike IgM, (e) RBD IgM, (f) Threshold displayed for IgM seropositive determination. Arrow = one PCR+ sample that displayed as IgG- but IgM+. All other PCR+ controls were IgG+ and IgM+/-.

a

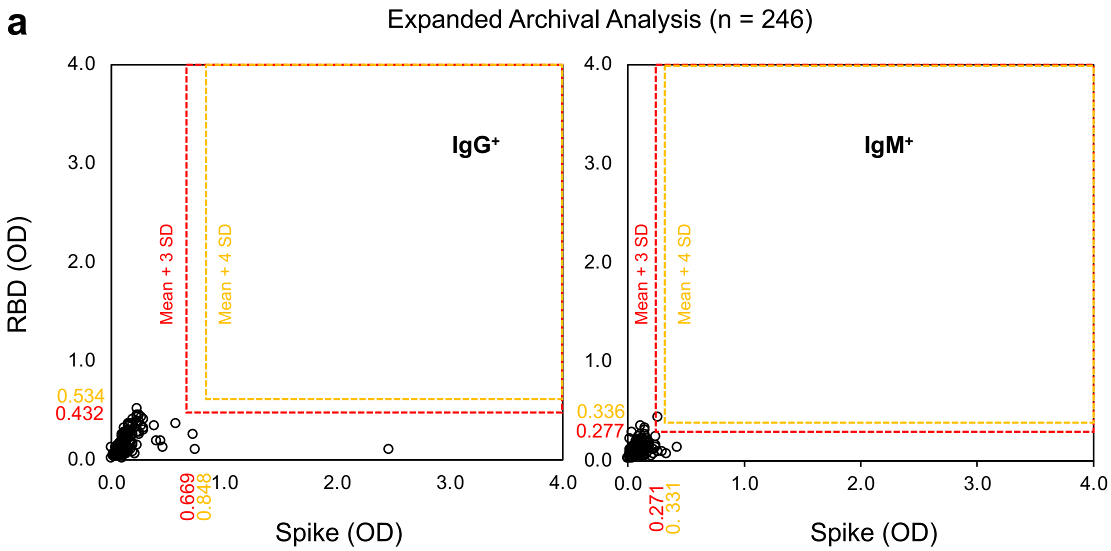

b

	Threshold	# of False Positives	Specificity
IgG	Mean + 3 StDev (OD = 0.669, 0.432)	0	100% (95% CI 98.5-100.0)
	Mean + 4 StDev (OD = 0.848,0.534)	0	100% (95% CI 98.5-100.0)
IgM	Mean + 3 StDev (OD = 0.271, 0.277)	1	99.59% (95% CI 97.8-100.0)
	Mean + 4 StDev (OD = 0.331, 0.337)	0	100% (95% CI 98.5-100.0)

Supplementary Figure 11: Specificity of semi-automated ELISA protocol. (a) Absorbance (OD) of 246 archival (pre-2019) samples for spike and RBD, IgG (left) and IgM (right). (b) Table displaying false positivity at a threshold of three or four standard deviations above the mean of the archival samples.

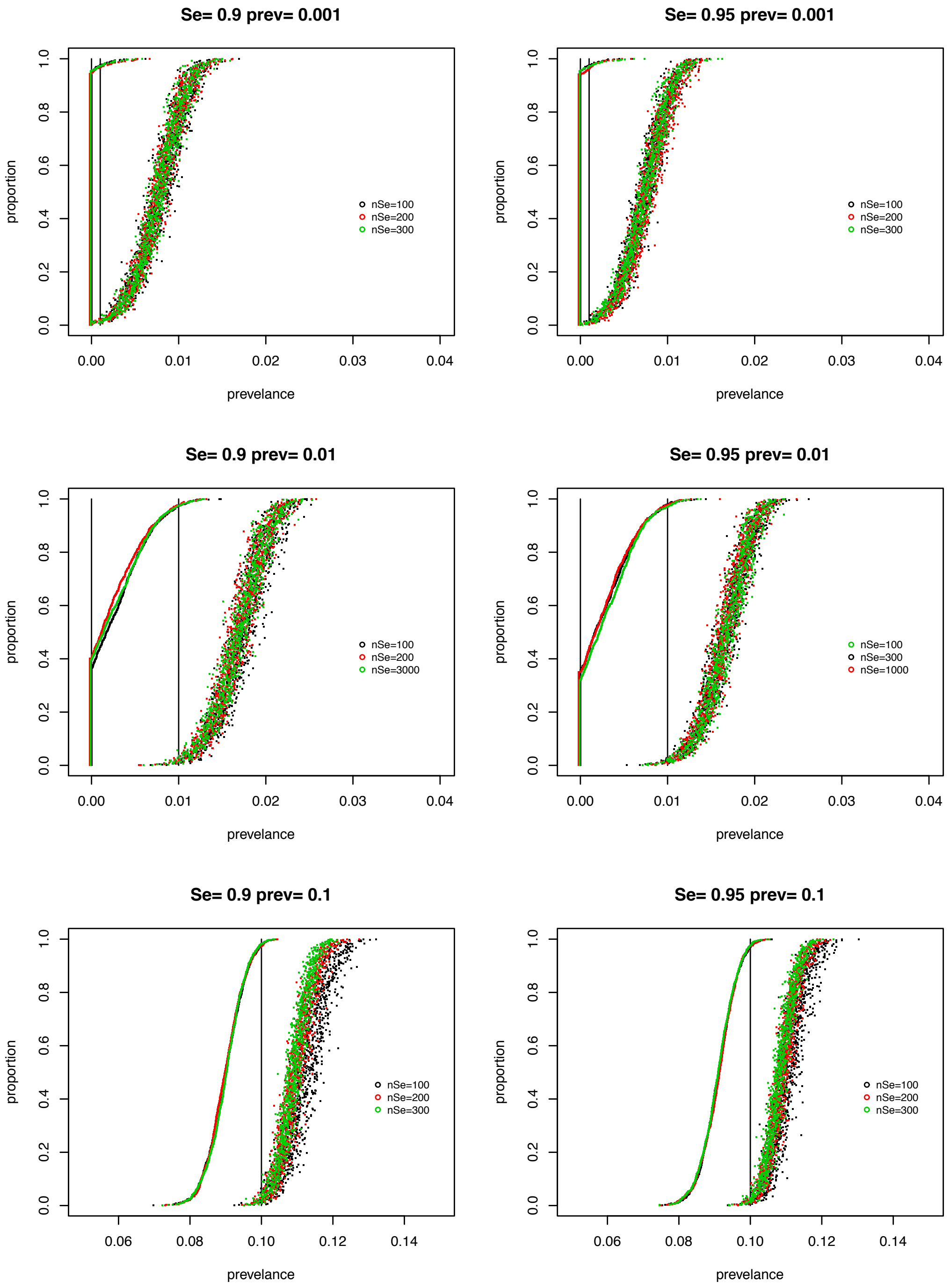

Supplementary Figure 12: Simulation and results of estimated 95% confidence intervals for serosurvey prevalence calculations. Each graph shows 95% CI's from 1000 replications of each condition. The CI's sorted by the lower bound. For graphs all graphs the true sensitivity is 0.95 and it is estimated with 1000 samples. The true specificity is either .90 or .95 and it is estimated with sample sizes of 100 (black), 300 (red), 300 (green). Each graph shows the different underlying true prevalence values of either 0.001, 0.01, or 0.1. The horizontal line shows the true prevalence for each simulation.

Semi-automated High-throughput ELISA Protocol (96-well Format)

Sequence	Parameter	Value	Description
1	Reagent	100 µL	RBD or Spike protein added by multichannel pipette*
2	Time	16 hr	4ºC incubation
3	Wash	3x 300 µL	PBST using Biotek EL406 96 wash head 1st and 2nd wash aspiration height = 3.68 mm above carrier, 3rd wash aspiration height = 3.43 mm above carrier, plus secondary aspiration 2.06 mm to left of well center same height
4	Reagent	200 µL	PBST + 5.0% milk using Biotek EL406 syringe dispense head
5	Time	2 hr	RT incubation stacked with lid on top plate
6	Wash	3x 300 µL	PBST using Biotek EL406 96 wash head 1st and 2nd wash aspiration height = 3.68 mm above carrier, 3rd wash aspiration height = 4.70 mm above carrier
7	Reagent	100 µL	Samples added by multichannel pipetter after tapping out remaining wash fluid, roughly 30 mL**
8	Time	1 hr	RT incubation stacked with lid on top plate
9	Wash	3x 300 µL	PBST using Biotek EL406 96 wash head, all 3 washes at aspiration height = 3.43mm above carrier, plus secondary aspiration 2.06 mm to left of well center at same height as well as 15 sec soak post each PBST addition
10	Reagent	100 µL	Secondary antibody IgG, IgM or IgA using Biotek EL406 syringe dispense head
11	Time	1 hr	RT incubation stacked with lid on top plate
12	Wash	3x 300 µL	PBST using Biotek EL406 96 wash head 1st and 2nd wash aspiration height = 3.68 mm above carrier plus secondary aspiration 2.06 mm to left of well center at same height, 25 sec soak for each, 3rd addition of PBST left in wells, plates restacked
13	Reagent	100 µL	Substrate added using EL406 syringe dispense head after aspirating using wash head at height = 3.43 mm above carrier plus secondary aspiration 2.06 mm to left of well center at same height
14	Time	10 min	RT incubation
15	Reagent	100 µL	Stop solution added using 2nd EL406 syringe dispense head
16	Detector	Absorbance	PHERAstar FSX end-point: 450/650 (BioTek Epoch2 Tested)

* optional use of peristaltic pump on Biotek EL406 for plate coating, dead volume increases protein consumption/sample
** optional use of 96 to 96 Integra transfer device to create multiple plates from deep-well plate

Supplementary Table 1: Assay table for semi-automated ELISA for SARS-CoV-2 seropositivity evaluation. Manual ELISA tested on PHERAstar FSX, automated setup validated on both PHERAstar FSX and BioTek Epoch2 plate readers. Epoch2 saturates at OD = 4, and any overflow values are post-hoc corrected to OD = 4 for calculations.

PROTEIN SEQUENCES OF FINAL PROTEINS

R619-X16: VRC-SARS-CoV-2 S-2P-3C-His8-Strep2

CVNLTTRTQLPPAYTNSFTRGVYYPDKVFRSSVLHSTQDLFLPFFSNVTWFHAIHVSGTNGTKRFDNPVLPFNDGVY
FASTEKSNIIRGWIFGTTLDSKTQSLLIVNNATNVVIKVCEFQFCNDPFLGVYYHKNNKSWMESEFRVYSSANNCTF
EYVSQPFLMDLEGKQGNFKNLREFVFKNIDGYFKIYSKHTPINLVRDLPQGFSALEPLVDLPIGINITRFQTLLALH
RSYLTPGDSSSGWTAGAAAYYVGYLQPRTFLLKYNENGTITDAVDCALDPLSETKCTLKSFTVEKGIYQTSNFRVQP
TESIVRFPNITNLCPFGEVFNATRFASVYAWNRKRISNCVADYSVLYNSASFSTFKCYGVSPTKLNDLCFTNVYADS
FVIRGDEVRQIAPGQTGKIADYNYKLPDDFTGCVIAWNSNNLDSKVGGNYNYLYRLFRKSNLKPFERDISTEIYQAG
STPCNGVEGFNCYFPLQSYGFQPTNGVGYQPYRVVVLSFELLHAPATVCGPKKSTNLVKNKCVNFNFNGLTGTGVLT
ESNKKFLPFQQFGRDIADTTDAVRDPQTLEILDITPCSFGGVSVITPGTNTSNQVAVLYQDVNCTEVPVAIHADQLT
PTWRVYSTGSNVFQTRAGCLIGAEHVNNSYECDIPIGAGICASYQTQTNSPGSASSVASQSIIAYTMSLGAENSVAY
SNNSIAIPTNFTISVTTEILPVSMTKTSVDCTMYICGDSTECSNLLLQYGSFCTQLNRALTGIAVEQDKNTQEVFAQ
VKQIYKTPPIKDFGGFNFSQILPDPSKPSKRSFIEDLLFNKVTLADAGFIKQYGDCLGDIAARDLICAQKFNGLTVL
PPLLTDEMIAQYTSALLAGTITSGWTFGAGAALQIPFAMQMAYRFNGIGVTQNVLYENQKLIANQFNSAIGKIQDSL
SSTASALGKLQDVVNQNAQALNTLVKQLSSNFGAISSVLNDILSRLDPPEAEVQIDRLITGRLQSLQTYVTQQLIRA
AEIRASANLAATKMSECVLGQSKRVDFCGKGYHLMSFPQSAPHGVVFLHVTYVPAQEKNFTTAPAICHDGKAHFPRE
GVFVSNGTHWFVTQRNFYEPQIITTDNTFVSGNCDVVIGIVNNTVYDPLQPELDSFKEELDKYFKNHTSPDVDLGDI
SGINASVVNIQKEIDRLNEVAKNLNESLIDLQELGKYEQGSGYIPEAPRDGQAYVRKDGEWVLLSTFLGRSLEVLFQ
GPGHHHHHHHHSAWSHPQFEKGGGSGGGGSGGSAWSHPQFEK

R619-X21: Kram-SARS-CoV-2 S-2P-His6

CVNLTTRTQLPPAYTNSFTRGVYYPDKVFRSSVLHSTQDLFLPFFSNVTWFHAIHVSGTNGTKRFDNPVLPFNDGVY
FASTEKSNIIRGWIFGTTLDSKTQSLLIVNNATNVVIKVCEFQFCNDPFLGVYYHKNNKSWMESEFRVYSSANNCTF
EYVSQPFLMDLEGKQGNFKNLREFVFKNIDGYFKIYSKHTPINLVRDLPQGFSALEPLVDLPIGINITRFQTLLALH
RSYLTPGDSSSGWTAGAAAYYVGYLQPRTFLLKYNENGTITDAVDCALDPLSETKCTLKSFTVEKGIYQTSNFRVQP
TESIVRFPNITNLCPFGEVFNATRFASVYAWNRKRISNCVADYSVLYNSASFSTFKCYGVSPTKLNDLCFTNVYADS
FVIRGDEVRQIAPGQTGKIADYNYKLPDDFTGCVIAWNSNNLDSKVGGNYNYLYRLFRKSNLKPFERDISTEIYQAG
STPCNGVEGFNCYFPLQSYGFQPTNGVGYQPYRVVVLSFELLHAPATVCGPKKSTNLVKNKCVNFNFNGLTGTGVLT
ESNKKFLPFQQFGRDIADTTDAVRDPQTLEILDITPCSFGGVSVITPGTNTSNQVAVLYQDVNCTEVPVAIHADQLT
PTWRVYSTGSNVFQTRAGCLIGAEHVNNSYECDIPIGAGICASYQTQTNSPASVASQSIIAYTMSLGAENSVAYSNN
SIAIPTNFTISVTTEILPVSMTKTSVDCTMYICGDSTECSNLLLQYGSFCTQLNRALTGIAVEQDKNTQEVFAQVKQ
IYKTPPIKDFGGFNFSQILPDPSKPSKRSFIEDLLFNKVTLADAGFIKQYGDCLGDIAARDLICAQKFNGLTVLPPL
LTDEMIAQYTSALLAGTITSGWTFGAGAALQIPFAMQMAYRFNGIGVTQNVLYENQKLIANQFNSAIGKIQDSLSST
ASALGKLQDVVNQNAQALNTLVKQLSSNFGAISSVLNDILSRLDPPEAEVQIDRLITGRLQSLQTYVTQQLIRAAEI
RASANLAATKMSECVLGQSKRVDFCGKGYHLMSFPQSAPHGVVFLHVTYVPAQEKNFTTAPAICHDGKAHFPREGVF
VSNGTHWFVTQRNFYEPQIITTDNTFVSGNCDVVIGIVNNTVYDPLQPELDSFKEELDKYFKNHTSPDVDLGDISGI

NASVVNIQKEIDRLNEVAKNLNESLIDLQELGKYEQYIKWPSGRLVPRGSPGSGYIPEAPRDGQAYVRKDGEWVLLS
TFLGHHHHHH

R619-X22: Kram-SARS-CoV-2 S-RBD(319-541)-His6

RVQPTESIVRFPNITNLCPFGEVFNATRFASVYAWNRKRISNCVADYSVLYNSASFSTFKCYGVSPTKLNDLCFTNV
YADSFVIRGDEVRQIAPGQTGKIADYNYKLPDDFTGCVIAWNSNNLDSKVGGNYNYLYRLFRKSNLKPFERDISTEI
YQAGSTPCNGVEGFNCYFPLQSYGFQPTNGVGYQPYRVVVLSFELLHAPATVCGPKKSTNLVKNKCVNFHHHHHH

R619-X24: Ragon-SARS-CoV-2 S-RBD(319-529)-3C-His8-SBP

RVQPTESIVRFPNITNLCPFGEVFNATRFASVYAWNRKRISNCVADYSVLYNSASFSTFKCYGVSPTKLNDLCFTNV
YADSFVIRGDEVRQIAPGQTGKIADYNYKLPDDFTGCVIAWNSNNLDSKVGGNYNYLYRLFRKSNLKPFERDISTEI
YQAGSTPCNGVEGFNCYFPLQSYGFQPTNGVGYQPYRVVVLSFELLHAPATVCGPKKGAGSSLEVLFQGPGSGSSHH
HHHHHHGGSGSSMDEKTTGWRGGHVVEGLAGELEQLRARLEHHPQGQREP

R619-X17: VRC-SARS-CoV S-2P-3C-His8-Strep2

SDLDRCTTFDDVQAPNYTQHTSSMRGVYYPDEIFRSDTLYLTQDLFLPFYSNVTGFHTINHTFGNPVIPFKDGIYFA
ATEKSNVVRGWVFGSTMNNKSQSVIIINNSTNVVIRACNFELCDNPFFAVSKPMGTQTHTMIFDNAFNCTFEYISDA
FSLDVSEKSGNFKHLREFVFKNKDGFLYVYKGYQPIDVVRDLPSGFNTLKPIFKLPLGINITNFRAILTAFSPAQDI
WGTSAAAYFVGYLKPTTFMLKYDENGTITDAVDCSQNPLAELKCSVKSFEIDKGIYQTSNFRVVPSGDVVRFPNITN
LCPFGEVFNATKFPSVYAWERKKISNCVADYSVLYNSTFFSTFKCYGVSATKLNDLCFSNVYADSFVVKGDDVRQIA
PGQTGVIADYNYKLPDDFMGCVLAWNTRNIDATSTGNYNYKYRYLRHGKLRPFERDISNVPFSPDGKPCTPPALNCY
WPLNDYGFYTTTGIGYQPYRVVVLSFELLNAPATVCGPKLSTDLIKNQCVNFNFNGLTGTGVLTPSSKRFQPFQQFG
RDVSDFTDSVRDPKTSEILDISPCAFGGVSVITPGTNASSEVAVLYQDVNCTDVSTAIHADQLTPAWRIYSTGNNVF
QTQAGCLIGAEHVDTSYECDIPIGAGICASYHTVSLLRSTSQKSIVAYTMSLGADSSIAYSNNTIAIPTNFSISITT
EVMPVSMAKTSVDCNMYICGDSTECANLLLQYGSFCTQLNRALSGIAAEQDRNTREVFAQVKQMYKTPTLKYFGGFN
FSQILPDPLKPTKRSFIEDLLFNKVTLADAGFMKQYGECLGDINARDLICAQKFNGLTVLPPLLTDDMIAAYTAALV
SGTATAGWTFGAGAALQIPFAMQMAYRFNGIGVTQNVLYENQKQIANQFNKAISQIQESLTTTSTALGKLQDVVNQN
AQALNTLVKQLSSNFGAISSVLNDILSRLDPPEAEVQIDRLITGRLQSLQTYVTQQLIRAAEIRASANLAATKMSEC
VLGQSKRVDFCGKGYHLMSFPQAAPHGVVFLHVTYVPSQERNFTTAPAICHEGKAYFPREGVFVFNGTSWFITQRNF
FSPQIITTDNTFVSGNCDVVIGIINNTVYDPLQPELDSFKEELDKYFKNHTSPDVDLGDISGINASVVNIQKEIDRL
NEVAKNLNESLIDLQELGKYEQGSGYIPEAPRDGQAYVRKDGEWVLLSTFLGRSLEVLFQGPGHHHHHHHHSAWSHP
QFEK

R619-X18: VRC-MERS-CoV S-2P-3C-His8-Strep2x2

YVDVGPDSVKSACIEVDIQQTFFDKTWPRPIDVSKADGIIYPQGRTYSNITITYQGLFPYQGDHGDMYVYSAGHATG
TTPQKLFVANYSQDVKQFANGFVVRIGAAANSTGTVIISPSTSATIRKIYPAFMLGSSVGNFSDGKMGRFFNHTLVL
LPDGCGTLLRAFYCILEPRSGNHCPAGNSYTSFATYHTPATDCSDGNYNRNASLNSFKEYFNLRNCTFMYTYNITED

EILEWFGITQTAQGVHLFSSRYVDLYGGNMFQFATLPVYDTIKYYSIIPHSIRSIQSDRKAWAAFYVYKLQPLTFLL
DFSVDGYIRRAIDCGFNDLSQLHCSYESFDVESGVYSVSSFEAKPSGSVVEQAEGVECDFSPLLSGTPPQVYNFKRL
VFTNCNYNLTKLLSLFSVNDFTCSQISPAAIASNCYSSLILDYFSYPLSMKSDLSVSSAGPISQFNYKQSFSNPTCL
ILATVPHNLTTITKPLKYSYINKCSRFLSDDRTEVPQLVNANQYSPCVSIVPSTVWEDGDYYRKQLSPLEGGGWLVA
SGSTVAMTEQLQMGFGITVQYGTDTNSVCPKLEFANDTKIASQLGNCVEYSLYGVSGRGVFQNCTAVGVRQQRFVYD
AYQNLVGYYSDDGNYYCLRACVSVPVSVIYDKETKTHATLFGSVACEHISSTMSQYSRSTRSMLKRRDSTYGPLQTP
VGCVLGLVNSSLFVEDCKLPLGQSLCALPDTPSTLTPASVGSVPGEMRLASIAFNHPIQVDQLNSSYFKLSIPTNFS
FGVTQEYIQTTIQKVTVDCKQYVCNGFQKCEQLLREYGQFCSKINQALHGANLRQDDSVRNLFASVKSSQSSPIIPG
FGGDFNLTLLEPVSISTGSRSARSAIEDLLFDKVTIADPGYMQGYDDCMQQGPASARDLICAQYVAGYKVLPPLMDV
NMEAAYTSSLLGSIAGVGWTAGLSSFAAIPFAQSIFYRLNGVGITQQVLSENQKLIANKFNQALGAMQTGFTTTNEA
FHKVQDAVNNNAQALSKLASELSNTFGAISASIGDIIQRLDPPEQDAQIDRLINGRLTTLNAFVAQQLVRSESAALS
AQLAKDKVNECVKAQSKRSGFCGQGTHIVSFVVNAPNGLYFMHVGYYPSNHIEVVSAYGLCDAANPTNCIAPVNGYF
IKTNNTRIVDEWSYTGSSFYAPEPITSLNTKYVAPQVTYQNISTNLPPPLLGNSTGIDFQDELDEFFKNVSTSIPNF
GSLTQINTTLLDLTYEMLSLQQVVKALNESYIDLKELGNYTYGSGYIPEAPRDGQAYVRKDGEWVLLSTFLGRSLEV
LFQGPGHHHHHHHHSAWSHPQFEKGGGSGGGGSGGSAWSHPQFEK

R619-X19: VRC-OC43-CoV S-2P-3C-His8-Strep2x2

VIGDLKCPLDSRTGSLNNIDTGPPSISTATVDVTNGLGTYYVLDRVYLNTTLFLNGYYPTSGSTYRNMALKGTDKLS
TLWFKPPFLSDFINGIFAKVKNTKVFKDGVMYSEFPAITIGSTFVNTSYSVVVQPRTINSTQDGVNKLQGLLEVSVC
QYNMCEYPHTICHPKLGNHFKELWHMDTGVVSCLYKRNFTYDVNATYLYFHFYQEGGTFYAYFTDTGVVTKFLFNVY
LGMALSHYYVMPLTCISRRDIGFTLEYWVTPLTSRQYLLAFNQDGIIFNAVDCMSDFMSEIKCKTQSIAPPTGVYEL
NGYTVQPIADVYRRKPDLPNCNIEAWLNDKSVPSPLNWERKTFSNCNFNMSSLMSFIQADSFTCNNIDAAKIYGMCF
SSITIDKFAIPNGRKVDLQLGNLGYLQSFNYRIDTTATSCQLYYNLPAANVSVSRFNPSTWNKRFGFIENSVFKPQP
AGVLTNHDVVYAQHCFKAPKNFCPCKLNSSLCVGSGPGKNNGIGTCPAGTNYLTCHNLCNPDPITFTGPYKCPQTKS
LVGIGEHCSGLAVKSDYCGGNPCTCQPQAFLGWSADSCLQGDKCNIFANLILHDVNSGLTCSTDLQKANTDIKLGVC
VNYDLYGISGQGIFVEVNATYYNSWQNLLYDSNGNLYGFRDYITNRTFMIRSCYSGRVSAAFHANSSEPALLFRNIK
CNYVFNNSLIRQLQPINYFDSYLGCVVNAYNSTAISVQTCDLTVGSGYCVDYSKNRRSRRAITTGYRFTNFEPFTVN
SVNDSLEPVGGLYEIQIPSEFTIGNMEEFIQTSSPKVTIDCAAFVCGDYAACKSQLVEYGSFCDNINAILTEVNELL
DTTQLQVANSLMNGVTLSTKLKDGVNFNVDDINFSSVLGCLGSECSKASSRSAIEDLLFDKVKLSDVGFVAAYNNCT
GGAEIRDLICVQSYKGIKVLPPLLSENQISGYTLAATSASLFPPWTAAAGVPFYLNVQYRINGLGVTMDVLSQNQKL
IANAFNNALDAIQEGFDATNSALVKIQAVVNANAEALNNLLQQLSNRFGAISSSLQEILSRLDPPEAEAQIDRLING
RLTALNAYVSQQLSDSTLVKFSAAQAMEKVNECVKSQSSRINFCGNGNHIISLVQNAPYGLYFIHFSYVPTKYVTAK
VSPGLCIAGDRGIAPKSGYFVNVNNTWMYTGSGYYYPEPITENNVVVMSTCAVNYTKAPYVMLNTSTPNLPDFREEL
DQWFKNQTSVAPDLSLDYINVTFLDLQVEMNRLQEAIKVLNGSGYIPEAPRDGQAYVRKDGEWVLLSTFLGRSLEVL
FQGPGHHHHHHHHSAWSHPQFEKGGGSGGGGSGGSAWSHPQFEK

R619-X20: VRC-HKU1-CoV S-2P-3C-His8-Strep2

VIGDFNCTNSFINDYNKTIPRISEDVVDVSLGLGTYYVLNRVYLNTTLLFTGYFPKSGANFRDLALKGSIYLSTLWY
KPPFLSDFNNGIFSKVKNTKLYVNNTLYSEFSTIVIGSVFVNTSYTIVVQPHNGILEITACQYTMCEYPHTVCKSKG
SIRNESWHIDSSEPLCLFKKNFTYNVSADWLYFHFYQERGVFYAYYADVGMPTTFLFSLYLGTILSHYYVMPLTCNA
ISSNTDNETLEYWVTPLSRRQYLLNFDEHGVITNAVDCSSSFLSEIQCKTQSFAPNTGVYDLSGFTVKPVATVYRRI
PNLPDCDIDNWLNNVSVPSPLNWERRIFSNCNFNLSTLLRLVHVDSFSCNNLDKSKIFGSCFNSITVDKFAIPNRRR
DDLQLGSSGFLQSSNYKIDISSSSCQLYYSLPLVNVTINNFNPSSWNRRYGFGSFNLSSYDVVYSDHCFSVNSDFCP
CADPSVVNSCAKSKPPSAICPAGTKYRHCDLDTTLYVKNWCRCSCLPDPISTYSPNTCPQKKVVVGIGEHCPGLGIN
EEKCGTQLNHSSCFCSPDAFLGWSFDSCISNNRCNIFSNFIFNGINSGTTCSNDLLYSNTEISTGVCVNYDLYGITG
QGIFKEVSAAYYNNWQNLLYDSNGNIIGFKDFLTNKTYTILPCYSGRVSAAFYQNSSSPALLYRNLKCSYVLNNISF
ISQPFYFDSYLGCVLNAVNLTSYSVSSCDLRMGSGFCIDYALPSSGGSGSGISSPYRFVTFEPFNVSFVNDSVETVG
GLFEIQIPTNFTIAGHEEFIQTSSPKVTIDCSAFVCSNYAACHDLLSEYGTFCDNINSILNEVNDLLDITQLQVANA
LMQGVTLSSNLNTNLHSDVDNIDFKSLLGCLGSQCGSSSRSLLEDLLFNKVKLSDVGFVEAYNNCTGGSEIRDLLCV
QSFNGIKVLPPILSETQISGYTTAATVAAMFPPWSAAAGVPFSLNVQYRINGLGVTMDVLNKNQKLIANAFNKALLS
IQNGFTATNSALAKIQSVVNANAQALNSLLQQLFNKFGAISSSLQEILSRLDPPEAQVQIDRLINGRLTALNAYVSQ
QLSDITLIKAGASRAIEKVNECVKSQSPRINFCGNGNHILSLVQNAPYGLLFIHFSYKPTSFKTVLVSPGLCLSGDR
GIAPKQGYFIKQNDSWMFTGSSYYYPEPISDKNVVFMNSCSVNFTKAPFIYLNNSIPNLSDFEAELSLWFKNHTSIA
PNLTFNSHINATFLDLYYEMNVIQESIKSLNSGRLEVLFQGPGGYIPEAPRDGQAYVRKDGEWVLLSTFLGHHHHHH
HHSAWSHPQFEK
